# A Seemingly Paradoxical Relationship Between Masturbation Frequency and Sexual Satisfaction

**DOI:** 10.1007/s10508-022-02305-8

**Published:** 2022-07-05

**Authors:** Nantje Fischer, Bente Træen

**Affiliations:** Department of Psychology, University of Oslo, Gaustadalleén 30, 0373 Oslo, Norway

**Keywords:** Masturbation, Solitary sexual activity, Sexual satisfaction, Intercourse activity, Pornography use, Body image

## Abstract

Despite many benefits related to masturbation, we know surprisingly little about how solo sex is associated with sexual satisfaction. Using questionnaire data from a probability-based sample of 4,160 Norwegians aged 18–89 years, we explored subgroups of women and men that differed in their masturbation–sexual satisfaction typology and examined whether sociodemographic, psychological, and sexual behavioral characteristics were associated with distinct masturbation–satisfaction patterns. A cluster analysis revealed four similar groupings for women and men, reflecting sex lives characterized by high masturbation/sexual satisfaction, low masturbation/sexual satisfaction, high masturbation/sexual dissatisfaction, or low masturbation/sexual dissatisfaction. While being younger, higher pornography consumption, and sexual variety were primarily associated with increased masturbation frequency, sexual distress and a negative body and genital self-image were more clearly associated with sexual dissatisfaction. Predicting different masturbation–satisfaction groupings also revealed some gender-specific findings in the use of pornography, and in the association between masturbation and intercourse frequency, which suggested a complementary pattern for women and a compensatory pattern for men. Our findings emphasize that the linkage between masturbation and sexual satisfaction warrants closer focus.

## Introduction

In many Western societies, masturbation is increasingly acknowledged for its multiple benefits, among others being a simple and safe sexual behavior, facilitating more pleasurable sexual practice and leading to greater body knowledge, sexual agency, and sexual self-esteem and fewer sexual difficulties (Carvalheira & Leal, 2013; Coleman, 2003; Dekker & Schmidt, 2003; Hensel & Fortenberry, 2014; Kontula & Haavio-Mannila, 2003; Rye & Meaney, 2007). In this study, we will define masturbation as autoerotic stimulation without the presence or involvement of another person (Kirschbaum & Pederson, 2018; Levesque, 2018).

Despite multiple benefits associated with masturbation (Coleman, 2003; Hensel & Fortenberry, 2014), we know surprisingly little about how sexual self-gratification is associated with sex life satisfaction. Sex life satisfaction or sexual satisfaction is a cognitive global evaluation where an individual evaluates the overall quality of their sexual life based on a self-selected standard (Neto, 2012). Although research has increasingly been interested in the study of sexual satisfaction, most studies only assess sex life satisfaction within a dyadic context (Byers & Rehman, 2014; Freihart et al., 2020). Hence, research on the association between masturbation and sexual satisfaction is limited and not well understood.

### Theoretical Framework

Studies have shown that the most commonly reported reasons for engaging in masturbation are feelings of sexual desire, sexual pleasure, and sexual satisfaction (Burri & Carvalheira, 2019; Carvalheira & Leal, 2013; Rowland et al., 2020). Based on these pleasure-oriented motives, it seems paradoxical that some studies find a negative relationship between masturbation and sexual satisfaction (Ayalon et al., 2019; Bancroft et al., 2011; DeLamater & Moorman, 2007; Lee et al., 2016; Rowland et al., 2020; Velten & Margraf, 2017) or satisfaction with sexual activity (Fischer et al., 2022). One overarching theory that may explain the negative association between masturbation and sex life satisfaction is sexual script theory (Gagnon & Simon, 2005). Sexual script theory suggests that all sexual practice and expression is scripted and determined by culture. Sexual scripts define when and how sexual behavior is “good” and “accepted” and when it is not (Gagnon & Simon, 2005; Wiederman, 2005). Thus, all sexual behavior is regulated by traditional, religious, and societal norms. According to the traditional sexual script, “good” sexuality is characterized by being heteronormative, partnered and legitimized by romantic love (Haus & Thompson, 2020; Træen & Lewin, 2008). Because masturbation occurs beyond those properties, it is often perceived as an uncomfortable issue that is less desirable and accepted than partnered sexuality (Kaestle & Allen, 2011).

Another characteristic of the traditional sexual script is that it is gendered (Wiederman, 2005). According to gendered scripts, men have a stronger and steadier interest in sex than women (Masters et al., 2013). Moreover, men discuss and compare their masturbatory behavior (Gagnon & Simon, 2005). Women, on the other hand, learn to express their sexuality within the context of committed relationships (Haus & Thompson, 2020; Wiederman, 2005), and their “experience of early masturbation seems often unconnected with any other domain of behavior” (Gagnon & Simon, 2005, p. 39). These gendered scripts have been reflected in recent research. A systematic review of qualitative studies (Onar et al., 2020) found that women sometimes seem to fear that masturbation reflects shortcomings within the relationship and worry that it would hurt a partner’s feelings. Other studies found that women often have more negative attitudes toward masturbation than men (Clark & Wiederman, 2000; Kaestle & Allen, 2011). Based on these gender-specific scripts, there are sound reasons to expect that the experience and level of sexual self-gratification may differ for women and men, which in turn may influence how women and men’s masturbation frequency is linked to their overall sexual satisfaction.

Because sexual self-gratification is often taboo in the social discourse and occurs without the presence of another person, the script for masturbation seems vague and ambiguous (Gagnon & Simon, 2005; Kirschbaum & Peterson, 2018). Moreover, masturbation is commonly surrounded by societal contradictions, for example, as being both stigmatized and promoted as a healthy sexual behavior (Kaestle & Allen, 2011; Watson & McKee, 2013). This implies that the social script for masturbation may likely vary across subcultures and individuals (Kirschbaum & Peterson, 2018). Indeed, several qualitative studies suggest a great variety of motives, meanings, and perceptions associated with masturbation (Hogarth & Ingham, 2009; Janssen et al., 2008; Onar et al., 2020). Experiences, for example, range from very negative to indifference, from perceiving masturbation as conflicted to feeling empowered; still others describe their masturbation as a perfunctory and predictable way to release sexual tension (Fahs & Frank, 2014; Hogarth & Ingham, 2009; Janssen et al, 2008; Kaestle & Allen, 2011).

Due to a lack of shared social script for masturbation (Kirschbaum & Peterson, 2018), we assume that there might be a broad variety in masturbation frequency that is, in turn, differently associated with sexual (dis)satisfaction. In the current study, we assess whether there are subgroups of individuals that differ in their masturbation frequency–sexual satisfaction typology. Moreover, if there are any subgroups, do relevant sociodemographic, psychological, and sexual behavioral factors predict distinct masturbation–satisfaction patterns? A literature review on masturbation and sexual satisfaction follows below.

### Sociodemographic Factors Associated with Masturbation and Sexual Satisfaction

Solo sexual activity has consistently been shown to decrease with increasing age (Corona et al., 2010; Fischer et al., 2022; Lee et al., 2016; Lindau et al., 2007; Palacios-Ceña et al., 2012; Schick et al., 2010a, 2010b). In the British National Surveys of Sexual Attitudes and Lifestyles (Natsal-3), 83% of men aged 16–24 years reported masturbating in the past four weeks (compared to 33% of men aged 65–74 years). The decline among women was 37% to 10%, respectively (Mercer et al., 2013). Apart from age, another important predictor of engaging in masturbation is a person’s relationship status. Most studies indicate that masturbation is more common among single adults than among those who are in a committed relationship (Burri & Carvalheira, 2019; DeLamater & Moorman, 2007; Regnerus et al., 2017; Rowland et al., 2020; Schick et al., 2010a, 2010b). As not being in a partnered relationship is associated with limited access to regular intercourse, single adults may compensate for this lack by frequent masturbation (Das et al., 2009; Regnerus et al., 2017). Access to partnered sex may also be central in explaining why partnered adults generally report higher sexual satisfaction than non-partnered adults (Dekker et al., 2020; Sánchez-Fuentes et al., 2014). In a representative population-based study of Norwegian emerging adults (Kvalem et al., 2019), being in a relationship was positively associated with sexual satisfaction in both genders. However, when controlling for covariates, especially intercourse frequency, the positive association between being partnered and sexual satisfaction diminished considerably among men and transformed into a negative association among women. This suggests that women without a partner are more sexually satisfied than partnered women, if one accounts for intercourse activity.

Studies that have explored associations between sexual identity and sexual satisfaction have been discrepant (Björkenstam et al., 2020; Byers & Rehman, 2014; Flynn et al., 2017; Kuyper & Vanwesenbeeck, 2011). Two Norwegian studies that compared different sexual behavioral groups found that women who have sex with women and men (WSWM; Træen et al., 2022b), and men who had sex exclusively with women (MSEW) reported being most satisfied with their sex life (Træen et al., 2022a).

### Psychological Factors Associated with Masturbation and Sexual Satisfaction

It is well-documented that individuals who feel favorably about their body appearance are more likely to be sexually satisfied (Woertman & van den Brink, 2012). Likewise, a more negative body and genital image have been associated with sexual dissatisfaction in women, and recently also in men (Holt & Lyness, 2007; Kaminsky-Bayer, 2020; Komarnicky et al., 2019; Schick et al., 2010a, 2010b; Træen et al., 2016; aan den Brink et al., 2018). A common mechanism that has been suggested is that body image concerns distract the individual from focusing on pleasurable stimuli during sexual activity, which over time lowers sexual satisfaction and sexual functioning (Barlow, 1986; Byers & Rehman, 2014; van den Brink et al., 2018). A presupposition for this rationale is that body self-consciousness and apprehensions are especially salient during partnered sexual activity (van den Brink et al., 2018). Interestingly, a recent study exploring the association between body image and orgasmic response among Norwegian women found that a more negative body image was not only linked to orgasmic difficulties during partnered sex but also during masturbation (Horvath et al., 2020).

Whether, or how, a positive body image and genital self-image are related to non-partnered sexual behaviors, such as masturbation, is largely understudied, especially in men (Kvalem et al., 2019). A few studies found a positive link between genital image and reported masturbation in women (Bowman, 2014; Herbenick et al., 2011). Other studies among women found that a more positive body image is related to higher masturbation frequency (Burri & Carvalheira, 2019; Shulman & Horne, 2003). Nevertheless, the relationship between body image and masturbation was relatively weak and based on low convenience samples. Whether body and genital image is related to men’s masturbation behavior remains to be explored.

### Sexual Behavior Associated with Masturbation and Sexual Satisfaction

It is reasonable to expect that women and men with broader sexual repertoires and openness to sexual experimentation have more active sex lives. For example, in a large representative sample of Australians aged 16–69 years, Richters et al. (2014) found that a greater variety of sexual practices and more sex partners were positively associated with having masturbated in the past year. Similarly, more erotic thoughts and fantasies, a general openness to new experiences, diverse sexual practices, a higher number of sex partners, and pornography use have all been linked to masturbation activity (Baćak & Štulhofer, 2011; Burri & Carvalheira, 2019; Carvalheira & Leal, 2013; Das, 2007; Richters et al., 2014). Indeed, all these factors might be intercorrelated, hence reflecting more sexualized personality patterns (Das, 2007; Das et al., 2009; Træen & Daneback, 2013).

Also, more sexual experimentation and variety is an important aspect of being sexually satisfied (Gillespie, 2017; Sánchez-Fuentes et al., 2014). In a recent study, Frederick et al. (2017) found a positive association between higher numbers of varied sexual acts (e.g., sex in unusual places, use of sex toys, watching pornography together, sex with several persons, varied sex positions) and sexual satisfaction among women and men. Pornography consumption, on the other hand, seems to have a negative association with sexual and relational satisfaction (Wright et al., 2017). In a survey of Norwegian adults aged 18–59 years, watching pornography while masturbating was inversely related to men’s sexual satisfaction, but unrelated to women’s sexual satisfaction (Træen & Daneback, 2013), a finding that is supported by recent reviews (Grubbs et al., 2019; Vaillancourt-Morel et al., 2019).

### The Linkage between Partnered and Solo Sex

Several models have been proposed to explain the links between solo and partnered sexual activity. According to the compensatory model, sexual self-stimulation functions as a substitute to channel sexual tension if dyadic sex is not possible, satisfying or less frequent than desired (Das et al., 2009; Regnerus et al., 2017). From this perspective, more intercourse activity is related to less masturbation (as masturbation is considered suboptimal and no longer necessary) and greater sexual satisfaction (as it is the preferred sexual act)—thus possibly causing a spurious negative relationship between masturbation and sexual satisfaction. Thus, if we find that frequent partnered sex is related to low masturbation frequency and high sexual satisfaction, this would support a compensatory pattern.

According to a complementary model, solo sexual activity is a supplement to, or reinforces, dyadic sexual activity (Regnerus et al., 2017). It is basically assumed that partnered sexual activity increases the need for more sexual activity (e.g., solo sex). Thus, if we find that more intercourse activity is related to high masturbation frequency and high sexual satisfaction, this would support a complementary pattern. Previous research indicates mixed evidence for the compensatory and complementary models (see Regnerus et al., 2017). While some studies found support for gender-specific models (Carvalheira & Leal, 2013; Fischer et al., 2022; Gerressu et al., 2008; Regnerus et al., 2017), other studies suggested bimodal patterns for women and men (Das, 2007; Das et al., 2009).

### Study Aim

To date, research on masturbation has been limited and only concentrated on linear processes linking masturbation with sexual satisfaction. Because there seems to be a lack of commonly shared instructions for masturbation (Kirschbaum & Peterson, 2018), it is reasonable to assume that people may rather fall into different masturbation-sexual satisfaction groupings and that there is not a linear relationship between masturbation frequency and sex life satisfaction. To close this gap, this study centered on two specific aims: (1) to explore subgroups of women and men that differ in their masturbation–sexual satisfaction profile, and (2) to assess whether sociodemographic, psychological, and sexual behavioral factors are associated with distinct masturbation–satisfaction patterns in Norwegian adults aged 18–89 years. Due to the exploratory focus of the study, no specific hypotheses are suggested.

## Method

### Participants and Procedure

Data in the current study were collected by Norsk Gallup, a subsidiary of Kantar, which is Norway's largest provider of analysis-based consultancy. About 40,000 individuals in Norway are members of Kantar’s Gallup Web Panel, and they are contacted regularly by e-mail to complete Web surveys. Potential participants for the Gallup Panel are randomly recruited via national phone registries (landline and mobile). As almost all households have a mobile or landline phone, and self-recruitment is not possible, the Web Panel is likely representative of Norway’s Internet population. A combination of small incentives and lotteries are provided, however, not large enough to account for the main factor in participation. Study participation for panel members is voluntary; members are never exposed to marketing or sales and ensured anonymity and safety. The ethical procedures comply with the Personal Data Act and the guidelines of the Norwegian Data Protection Authority, as well as follow the standards of Norway's Market Research Association and The European Society for Opinion and Market Research.

For the current study, in March 2020, e-mails were sent to a randomly recruited sample of 11,685 individuals from the Web Panel. In total, 4,160 adults aged 18–89 years filled out the questionnaire, which resulted in a response rate of 36%. Fifty-one percent filled out the questionnaire on their mobile. The overall survey aimed to collect important data on sexual experiences and attitudes and to produce comprehensive knowledge about sexual health in Norway. Survey questions were for example: Background characteristics (e.g., gender, education, religion, place of residence, sexual orientation, civil status, self-estimated general health, BMI, and body image), and various sexual experiences and habits (e.g., extradyadic activity, use of protection, sexual attitudes, pornography use, sexting, sexual satisfaction, sexual activity, and sexual problems). The research questions for this study were planned after the data were collected, but before conducting the statistical analyses. All questions used in this study were based on predefined checkboxes (the respective response options are shown under Measures).

Sociodemographic and sexual behavior characteristics are depicted in Table 1. On average, men were older than women (*M*_women_ = 44.4, SD = 16.85; *M*_men_ = 48.4, SD = 17.09, *t*_(4146)_ = 7.61, *p* < 0.001; *η*2 = 0.01). Most women (67%) and men (62%) reported some form of university education (e.g., Bachelor’s degree or similar, Master’s degree, Ph.D. or similar). While six in ten (60%) said that they had no religious affiliation, those reporting being religious were for the most part either Christians with no specific denomination (18%) or Protestants (17%). More than half of the women (60%) and men (54%) reported living in urban areas, and about one in four in a small town. The proportion of partnered participants was somewhat lower in women (71%) than men (77%).

**Table 1:** Sociodemographic characteristics of women and men in Norway (percent)

	Women (*n* = 1967)		Men (*n* = 2181)		*p*	Effect size^*a*^
	*%*	*n*	*%*	*n*		
Age groups					< .001	.14
< 30 years	26.0	511	17.0	370		
30–39 years	20.7	408	19.7	430		
40–49 years	14.4	283	15.5	339		
50–59 years	15.8	310	18.8	411		
60–69 years	13.7	269	13.3	289		
70 + years	9.5	186	15.7	342		
Level of education					< .001	.07
6–8 years of schooling	0.4	7	1.2	25		
9–10 years of schooling	4.0	78	5.3	114		
12–13 years of schooling	28.5	559	32.1	696		
Lower university level	43.8	858	39.2	851		
Higher university level	23.3	456	22.3	484		
Religious					.361	.04
No	60.0	1145	59.0	1264		
Christian—no denomination	18.2	348	18.0	385		
Roman Catholic	0.8	16	0.7	14		
Protestant	16.7	318	17.3	371		
Baptist/Methodist/Evangeline	2.2	42	3.4	72		
Islam/Muslim	0.8	15	0.6	12		
Other	1.2	23	1.1	24		
Place of residence					< .001	.06
Urban/city	60.0	1174	54.0	1173		
Small town	25.3	496	28.2	612		
Rural	14.7	287	17.8	387		
Sexual orientation					< .001	.09
Heterosexual	94.5	1823	92.7	1983		
Homosexual/lesbian	1.1	22	3.9	83		
Bisexual/pansexual	3.7	72	2.8	60		
Asexual/other	0.6	12	0.7	14		
Relationship status					< .001	.10
Partnered^b^	71.0	1392	76.6	1663		
Unmarried^c^	18.4	361	18.0	390		
Separated/divorced^c^	7.4	145	4.3	93		
Widow/widower^c^	3.2	62	1.1	24		
Ever intercourse^d^						
Yes	95.2	1811	93.4	1990	.015	-.04
No	4.8	91	6.6	141		

Chi-square test of differences for gender

^a^phi coefficient for 2 by 2 tables, Cramer’s V for tables lager than 2 by 2; ^b^married/cohabitant/registered partnership or in a current permanent relationship; ^c^and being single/currently not in a permanent relationship; ^d^vaginal, oral or anal intercourse

When asked about sexual orientation, 95% of women and 93% of men reported being heterosexual. In addition, around 3% reported being bisexual or pansexual, and under 1% asexual/other. The vast majority reported having experienced sexual intercourse (vaginal, oral, or anal).

### Measures

The majority of the measures in this study were retrieved from previous Nordic and Norwegian surveys (Kvalem et al., 2014; Lewin et al., 2000; Traeen & Stigum, 2010; Træen et al., 2019), the British National Survey of Sexual Attitudes and Lifestyles (Natsal-3; Mitchell et al., 2013), and the German Health and Sexuality Survey (GeSiD; Matthiesen et al., 2021). The measures indexing the frequency of masturbation and intercourse were taken from the Sexual Relationships and Activities Questionnaire (SRA-Q), a questionnaire modified from previous validated measures and evaluated for its face validity (Lee et al., 2016). The same measures were also used in the multinational survey on healthy sexual aging (Norway, Denmark, Belgium, and Portugal) where the items developed in English were translated into local languages (e.g., Norwegian) using the translation and back translation method. To assess distress related to one or more sexual difficulties, we used the Natsal-SF instrument, which is a reliable and valid measure of sexual functioning (Mitchell et al., 2012). This measure was also used in the multinational survey on healthy sexual aging where a translation and back translation method was applied (English, Norwegian). Individuals’ sociodemographic backgrounds were mainly assessed by taking measures from the GeSiD survey, a questionnaire that has been thoroughly piloted and tested (Matthiesen et al., 2021), and the Healthy Sexual Aging project (Træen et al., 2019). In some instances, the original wording had to be slightly modified, and all questions used in this study were previously piloted on a self-selected Facebook sample.

Masturbation frequency was assessed by a question previously used in the English Longitudinal Study of Ageing (ELSA; Lee et al., 2016): “How often did you masturbate in the past month?” Responses were made on a 7-point scale ranging from 1 = none to 7 = more than once a day (see Table 2).

**Table 2:** Frequency of masturbation during the past month in Norwegian women and men, by age groups (percent)

Masturbation frequency	All	< 30 years	30–39 years	40–49 years	50–59 years	60–69 years	70 + years	*p*	Eta squared
*Women*									
None	33.7	22.5	26.4	29.7	40.3	46.0	59.4	< .001	.97
Once	18.5	16.9	16.2	16.3	20.7	23.0	21.3		
2 or 3 times	24.4	26.3	26.6	27.4	24.8	20.6	13.8		
Once a week	10.1	15.3	10.2	11.8	6.9	6.3	3.1		
2 or 3 times a week	12.0	16.1	19.3	13.7	6.9	3.6	2.5		
Once a day	1.1	2.5	0.8	1.1	0.3	0.4	0.0		
More than once a day	0.2	0.4	0.5	0.0	0.0	0.0	0.0		
*n* =	1820	472	383	263	290	252	160		
*Men*									
None	15.8	4.8	5.1	6.4	14.3	27.9	42.7	< .001	.97
Once	10.1	4.5	5.4	6.4	10.7	13.2	22.5		
2 or 3 times	19.1	15.2	14.0	19.3	21.0	29.4	19.0		
Once a week	15.1	14.6	17.9	16.2	19.9	11.8	7.9		
2 or 3 times a week	29.2	40.3	40.9	36.4	28.4	15.4	7.0		
Once a day	8.7	17.5	13.0	12.5	4.3	1.8	0.6		
More than once a day	2.0	3.1	3.7	2.8	1.3	0.4	0.3		
*n* =	2069	355	408	327	391	272	316		

One-way ANOVA testing for group differences

Sexual satisfaction was indicated by asking: “All things considered – how satisfied are you with your sexual life?” Responses were provided on a 5-point scale ranging from 1 = very dissatisfied to 5 = very satisfied.

Gender was measured by asking: “Are you: 1 = man, 2 = woman, 3 = other (please describe how you identify yourself).” In total, 12 individuals chose “other.” Two identified as trans men, one as non-binary, and nine wrote down their age and not a description of their gender. It was decided to exclude those 12 participants from the analysis.

Age was measured by year of birth.

Relationship status was indexed via two questions: 1) “What is your marital status?” with scores 1 = unmarried, 2 = separated/divorced, 3 = widow/widower, 4 = married/cohabitant/registered partnership; and 2) “If unmarried, separated/divorced, or widow/widower: Are you currently in a permanent relationship?” with response categories 1 = no, 2 = yes, with one person, 3 = yes, with several persons.

Level of education was measured by asking (Træen et al., 2019) “What is your highest level of formal education?” with responses ranging from 1 = primary school (6–8 years education) to 5 = higher university level (Master’s degree, Ph.D. or similar).

Religious affiliation was index by the following question (Træen et al., 2019): “Do you currently regard yourself as belonging to any particular religion?” Response options were 1 = no, 2 = yes, Christian—no denomination, 3 = yes, Roman Catholic, 4 = yes, Protestant, 5 = yes, Baptist/Methodist/Evangelical, 6 = yes, Islam/Muslim, and 7 = yes, other. Scores were recoded with 0 = no religious affiliation (1), and 1 = some religious affiliation (2–7).

Level of distress related to one or more sexual difficulties was indexed via seven items for each gender (“lacked interest in having sex,” “lacked enjoyment in sex,” “felt anxious during sex,” “felt no excitement or arousal during sex,” “did not reach climax (experienced an orgasm) or took a long time to reach climax despite feeling excited/aroused,” “reached climax more quickly than I would have liked,” “if woman: had an uncomfortably dry vagina,” and “if man: had trouble getting or keeping an erection”). These measured the level of distress about the respective sexual difficulty. All items were rated on a 4-point scale (1 = no distress, 2 = mild distress, 3 = moderate distress, and 4 = severe distress) and summed into a composite indicator, with higher composite scores indicating greater sexual distress. The measures were adapted from the British Natsal-3 survey (Mitchell et al., 2013).

Body image was measured by a single item previously used in Frederick et al. (2016), namely “How dissatisfied or satisfied are you with your physical appearance.” Genital self-image was indexed via the following question (Kvalem et al., 2014): “How dissatisfied or satisfied are you with the appearance of your genitalia (e.g., penis/labia)?” Both items were rated on a 7-point scale (1 = extremely dissatisfied to 7 = extremely satisfied), which was subsequently recoded so that higher scores indicated greater dissatisfaction.

Indicators on the number of sexual partners over a lifetime were adapted from the German GeSiD Survey, 2019 (https://gesid.eu/studie/). Two single-item measures, “In your lifetime, how many men have you had vaginal, oral or anal intercourse with,” and “In your lifetime, how many women have you had vaginal, oral or anal intercourse with,” were used to calculate the total number of sexual partners over a lifetime.

Frequency of pornography use was measured by a single-item measure (adapted from GeSiD; https://gesid.eu/studie/), namely “How often have you seen pornography in the last 12 months?” scaled from 1 = never to 8 = daily.

Sexual variety was assessed using the following stem (Dekker & Matthiesen, 2015): “What have you tried, or want to try, during sex?” This was followed by seven different sexual activities: “watch pornography together,” “have sex in unusual places,” “have sex with several persons at the same time,” “use sex toys (e.g., dildo or vibrator),” “role play,” “BDSM (sadomasochism, bondage, dominance and submission),” and “have sex in a swingers-club or swap partners.” Response options (1 = I have already tried it, 2 = I want to try it, and 3 = I have not, and do not, want to try it) were recoded into 1 = tried/wish to try (1 + 2), and 2 = have not/do not want to try. An accumulated number of sexual experiences and desires was calculated, which ranged from 0 = I have not, and do not want to try any of those activities to 7 = I have tried/want to try all of those activities.

Intercourse frequency was measured by an item adapted from the British ELSA survey (Lee et al., 2016): “How many times have you had sexual intercourse (vaginal, anal, or oral sex) during the last month?” Responses options were 1 = no times, 2 = once in the past month, 3 = 2 or 3 times the past month, 4 = once a week, 5 = 2 or 3 times per week, 6 = once a day, and 7 = more than once a day.

### Statistical Analysis

A two-step cluster analysis was used to identify subgroups of individuals representing different levels of masturbation frequency and sexual satisfaction. The two-step cluster analysis is an exploratory technique recommended for large data that incorporates an algorithm based on two distinct step sets (Norušis, 2012; Tkaczynski, 2017). First, to decrease the size of the matrix (the distance between all objects to be clustered), cases were arranged into pre-clusters by an algorithm (Norušis, 2012). The algorithm chooses, dependent on the distance measures (here: log-likelihood), whether an observed case is added to a previously formed pre-cluster or forms a new pre-cluster. This process is called pre-clustering. Second, the pre-clusters are arranged into groups utilizing hierarchical clustering algorithms (Bayesian Information Criterion (BIC)). This stage is called clustering. The validity of the cluster solution is assessed using the average silhouette measure, which indicates the quality of the clustering by determining how well cases in a cluster are similar (small within-cluster distance), while the distance between clusters is large (Norušis, 2012). The measure varies between − 1 and 1 and should be above 0.2 to ensure an adequate separation distance between divergent clusters (Tkaczynski, 2017). The importance of the variables is evaluated by a score that indexes the variables predictive importance in the cluster formation, preferably above 0.02 (Tkaczynski, 2017). Initially, we conducted a cluster analysis including the total sample. However, gendered scripts for masturbation give reason to believe that masturbation activity in women is differently associated with sexual satisfaction than in men. Moreover, because women and men vary substantially in how much they masturbate (see Table 2), relatively high levels of masturbation in women may not necessarily reflect relatively high levels of masturbation in men, but rather medium/low masturbation frequency. A shared-cluster solution therefore seemed indefinite.

Subsequently, gender-specific multinomial logistic regression analyses were carried out to explore how selected sociodemographic, psychological, and sexual behavioral factors are associated with different cluster memberships. Findings are displayed as adjusted odds ratios (AOR) with 95% confidence intervals (CI) with “high masturbation frequency and high sexual satisfaction” as the reference category/cluster. The group labels do not reflect an absolute level of high/low masturbation and sexual (dis)satisfaction, but rather the relatively greater or lesser degree of masturbation frequency/sexual satisfaction compared to the other clusters. All analyses were conducted using IBM SPSS version 27.0.

## Results

The majority of women (66%) and men (84%) reported masturbation in the past month (Table 2). Most women reported that they had masturbated 2 or 3 times per month, whereas masturbating 2 or 3 times per week was most common for men. Among both women and men, there was a statistically significant difference in masturbation frequency across age groups. Women between 18 to 49 years showed a similar pattern, with about 26% masturbating 2 or 3 times per month. However, across age groups there was a general increase among women reporting no masturbation. At ages 70 years and older, more than half (59%) of the women said they had not masturbated during the preceding month. The proportion of men reporting masturbation 2–3 times per week decreased with increasing age from 40% in the youngest age groups (18–30 years), to 7% among those aged 70 + years.

### Cluster Analysis of Masturbation Frequency–Sexual Satisfaction Patterns

Conducting a two-step cluster analysis on the whole sample—using the variables masturbation frequency and sexual satisfaction—revealed a theoretically meaningful grouping of four clusters. The largest cluster (33.1%) was characterized by high masturbation frequency (*M* = 4.5; SD = 0.93) and high sexual satisfaction (*M* = 3.8; SD = 0.71) and was called *High masturbation and Satisfied* (HmS). The smallest cluster (16.7%) included women and men characterized by low masturbation frequency (*M* = 1.4; SD = 0.49) and low sexual satisfaction (*M* = 2.4; SD = 0.74). This cluster was labeled *Low/no masturbation and Dissatisfied* (LmD). A third cluster (31.5%) contained participants with low masturbation frequency (*M* = 1.9; SD = 0.87) but high sexual satisfaction (*M* = 4.4; *SD* = 0.48); this was called *Low/no masturbation and Satisfied* (LmS). The fourth cluster (18.7%) was characterized by a high masturbation frequency (*M* = 4.4; SD = 1.1) and low sexual satisfaction (*M* = 1.7; SD = 0.47), and subsequently called *High masturbation and Dissatisfied* (HmD).

Because of the substantial gender differences in masturbation frequency and gendered social scripts for masturbation, the cluster analysis was repeated separately for women and men, which resulted in a three-cluster solution for the gendered subsamples. For both genders, the three-cluster solution revealed an indistinct grouping, where masturbation frequency varied over vast areas of the scale (Men: 1 = no masturbation to 7 = more than once a day; women: 1 = no masturbation to 5 = 2 or 3 times a week). The other two clusters were similar to those found in the total sample. Because the three-cluster solution contained one ambiguous cluster (masturbation scores varied over vast areas of the scale), the clustering was rerun with a manually fixed number of four clusters. The four-cluster solution contained no indistinct cluster was theoretically meaningful and offered face-valid solutions in both subsamples. Compared to the three-cluster solution, the final clustering (four) did not decrease the average silhouette measure (0.50 = implicating fair to good clustering). Masturbation frequency and sexual satisfaction were equally important for the cluster formation, with a predictor importance of 1.0 for both variables. A cluster membership variable was created for women and men. More details about the clustering (outputs and syntax) can be found elsewhere (Fischer, 2022). Characteristics of the gender-specific four-cluster solution are shown in Table 3.

**Table 3:** Subgroups of individuals that represent different combinations of masturbation frequency and sexual satisfaction, separately for women and men

Clusters		Women (%)				Men (%)			
		1	2	3	4	1	2	3	4
		High M satisfied	Low/no M satisfied	High M dissatisfied	Low/no M dissatisfied	High M Satisfied	Low/no M satisfied	High M dissatisfied	Low/no M dissatisfied
		25.6	40.7	21.6	12.0	35.8	27.9	16.5	19.8
*n*		445	707	375	209	731	570	336	405
		*M(SD)*				*M(SD)*			
Masturbation frequency ^a^		3.9 (.95)	1.4 (.48)	3.8 (.92)	1.7 (.72)	4.9 (.73)	1.9 (.88)	5.3 (.70)	2.6 (.90)
Sexual satisfaction^b^		4.3 (.47)	3.9 (.76)	2.4 (.63)	1.6 (.50)	3.9 (.67)	4.2 (.63)	1.6 (.50)	2.2 (.69)

M = masturbation frequency during the past month; Cluster 1 = HmS, cluster 2 = LmS, cluster 3 = HmD, cluster 4 = LmD

^a^ 1 = none, 2 = once, 3 = 2 or 3 times, 4 = once a week, 5 = 2 or 3 times a week, 6 = once a day, 7 = more than once a day;

^b^ 1 = very dissatisfied, 2 = somewhat dissatisfied, 3 = neither satisfied nor dissatisfied, 4 = quite satisfied, and 5 = very satisfied

### Characteristics of Women’s and Men’s Cluster Affiliation

Except for education in women, there were statistically significant differences in all sociodemographic, psychological, and sexual behavioral factors by cluster (Table 4). However, only age, frequency of pornography use, number of sexual experiences/desires, and intercourse frequency had large effect sizes.

**Table 4:** An overview of the sociodemographic, psychological, and sexual behavioral factors of women and men in each cluster

	Women						Men							
	1	2	3	4	*p*	Effect size	1	2	3	4	*p*	Effect size	Min	Max
	HmS	LmS	HmD	LmD			HmS	LmS	HmD	LmD				
Age	38.1	48.9	41.3	46.1	< .001	.08	41.4	54.7	42.8	57.7	< .001	.19	18	87
Education														
6–8 years of schooling	0.0	0.0	0.0	0.5	.144	.06	0.4	1.2	0.3	2.1	.007	.07		
9–10 years of schooling	2.1	3.6	2.6	5.1			3.2	6.2	3.9	6.4				
12–13 years of schooling	25.6	27.1	28.9	30.5			32.7	34.2	30.3	28.5				
Lower university level	49.0	45.6	43.6	39.1			39.2	37.7	41.4	44.3				
Higher university level	23.3	23.7	24.9	24.9			24.6	20.8	24.0	18.7				
Religious					< .001	.18					< .001	.20		
Yes	29.1	47.2	28.9	34.4			31.6	50.5	27.2	46.9				
No	70.9	52.8	71.1	65.6			68.4	49.5	72.8	53.1				
Relationship status					< .001	.18					< .001	.19		
Partnered^a^	80.5	84.4	54.3	64.5			77.6	92.90	55.9	83.5				
Unmarried^b^	12.6	6.8	33.1	21.8			16.7	4.2	36.8	9.1				
Separated/divorced^b^	6.6	5.0	9.7	11.2			5.1	1.7	6.6	5.9				
Widow/widower^b^	0.2	3.8	2.9	2.5			0.6	1.1	0.7	1.6				
Distress about sexual difficulties^c^	2.4	2.3	2.8	3.0	< .001	.08	2.3	2.4	2.8	2.6	< .001	.04	1	4
Body image^d^	3.1	3.4	3.7	3.8	< .001	.04	3.1	3.0	3.7	3.5	< .001	.06	1	7
Genital self-image^d^	2.8	3.2	3.5	3.7	< .001	.04	2.9	3.0	3.5	3.6	< .001	.05	1	7
Number of sex partners (lifetime)^e^	14.4	8.7	14.2	11.4	< .001	.02	19.9	14.9	15.7	17.0	.005	.01	0	200
Frequency of pornography use^e^	3.3	2.0	2.9	2.3	< .001	.10	6.0	3.4	6.5	4.2	< .001	.34	1	8
Number of sexual experience/desires^e^	3.3	2.0	3.2	2.4	< .001	.12	3.6	2.2	3.7	2.3	< .001	.13	0	7
Intercourse frequency^f^	3.5	2.9	1.7	1.6	< .001	.23	3.1	3.3	1.6	1.8	< .001	.24	1	7

Percentage given for education, religiosity, and relationship status; mean scores given if not otherwise specified

^a^married/cohabitant/registered partnership or in a current permanent relationship; ^b^and being single/currently not in a permanent relationship ^c^higher scores denote greater sexual distress ^d^higher scores denote greater dissatisfaction ^e^higher scores denote higher frequency/number; Max value for number of sex partners (lifetime) varied across gender (women = 149); One-way ANOVA (Effect size = eta squared) and Chi-square test of differences among clusters (Effect size = phi coefficient for 2 by 2 tables, Cramer’s V for tables lager than 2 by 2)

### Predictors of the Masturbation and Satisfaction Clusters in Women

#### Sociodemographic Factors

Findings from the multinomial logistic regression analysis on cluster membership for women are shown in Table 5. Among women, being older increased the odds of reporting no or low masturbation and high sexual satisfaction (AOR = 1.03) compared with the reference cluster (HmS). Separated/divorced women were more likely to report high masturbation and being sexually satisfied (HmS) than those belonging to any other cluster (AOR _LmS_ = 0.20; AOR _HmD_ = 0.29; AOR _LmD_ = 0.17). Women with higher education were more likely to report high masturbation and being sexually dissatisfied (AOR _HmD_ = 1.51) than the reference group (HmS), suggesting that higher education is related to less sexual satisfaction, but not necessarily less masturbation.

**Table 5:** Predictors of the three masturbation and satisfaction clusters (LmS, HmD, LmD) in comparison with the reference group (HmS: High masturbation and Satisfied) in women (*n* = 677)

	Low/no masturbation Satisfied (LmS)		High masturbation Dissatisfied (HmD)		Low/no masturbation Dissatisfied (LmD)	
	AOR	95% CI	AOR	95% CI	AOR	95% CI
Sociodemographic characteristics						
Age	1.03^**^	1.01–1.05	1.02	0.99–1.04	1.00	0.98–1.03
Education	1.22	0.92–1.63	1.51^*^	1.10–2.08	1.26	0.87–1.84
Religious	0.80	0.48–1.31	1.01	0.55–1.85	1.02	0.51–2.07
Relationship status^a^						
Unmarried^b^	0.47	0.20–1.13	1.30	0.59–2.89	0.93	0.36–2.36
Separated/divorced^b^	0.20^**^	0.07–0.60	0.29^*^	0.10–0.81	0.17^**^	0.05–0.65
Widow/widower^b^	0.13	0.01–1.63	0.22	0.01–4.09	0.36	0.02–7.18
Psychological factors^c^						
Distress about sexual difficulties	1.11	0.88–1.41	1.87^***^	1.39–2.44	2.53^***^	1.83–3.51
Body image	1.12	0.92–1.37	1.27^*^	1.01–1.59	1.38^*^	1.06–1.79
Genital self-image	1.18	0.99–1.40	1.18	0.98–1.43	1.10	0.88–1.38
Sexual behaviour^d^						
Number of sex partners (lifetime)	0.99	0.98–1.00	0.99	0.98–1.01	0.99	0.97–1.01
Frequency of pornography use	0.79^***^	0.69–0.91	0.96	0.82–1.12	0.79^*^	0.65–0.95
Number of sexual experiences/desires	0.82^*^	0.70–0.96	1.16	0.97–1.39	0.95	0.77–1.18
Intercourse frequency	0.72^***^	0.60–0.86	0.36^***^	0.29–0.46	0.32^***^	0.24–0.43

^*^p < 0.05. ^**^p < 0.01. ^***^p < 0.001;

^a^ Reference category = married/cohabitant/registered partnership or in a current permanent relationship; ^b^ and currently not in a permanent relationship; ^c^ higher scores denote greater sexual distress/greater dissatisfaction; ^d^ higher scores denote higher frequency/numbers

#### Psychological Factors

Compared with the reference cluster (HmS), women who experienced distressing sexual problems were more likely to be dissatisfied with their sex life (AOR _HmD_ = 1.87; AOR _LmD_ = 2.53). Similarly, a more negative body image was associated with being sexually dissatisfied (AOR _HmD_ = 1.27; AOR _LmD_ = 1.38), compared to participants in the reference cluster (HmS).

#### Sexual Behavioral Factors

Women who reported frequent pornography use were less likely to fall into clusters characterized by no or low masturbation frequency (AOR _LmS/LmD_ = 0.79) than the reference cluster (HmS). Women with greater sexual variety were more likely to report high masturbation and sexual satisfaction (HmS) than no or low masturbation and satisfaction (AOR _LmS_ = 0.82). With respect to partnered sex, women with higher intercourse frequency were more likely to report high masturbation and satisfaction (HmS) than any other group (AOR _LmS_ = 0.72; AOR _HmD_ = 0.36; AOR _LmD_ = 0.32).

### Predictors of the Masturbation and Satisfaction Clusters in Men

#### Sociodemographic Factors

Table 6 shows the findings of a multinomial logistic regression analysis investigating predictors of the three clusters (LmS, HmD, LmD) in comparison with the reference cluster (high masturbation and satisfied; HmS) in men. With increasing age, men were significantly more likely to fall into the clusters characterized by low masturbation frequency (AOR _LmS_ = 1.04; AOR _LmD_ = 1.03) than men in the reference cluster (HmS). Unmarried men were more likely to report high masturbation and being sexually satisfied than no or low masturbation and sexually dissatisfied (AOR _LmD_ = 0.21). Separated/divorced men were more likely to report high rather than low masturbation frequency (AOR _LmS_ = 0.33). Conversely, those who were married/cohabitant or in a registered partnership were more frequently characterized by LmS, HmD, LmD than HmS. Men with higher education were more likely to report high masturbation and being sexually dissatisfied (AOR _HmD_ = 1.34) than the reference group (HmS), suggesting that higher education is related to less sexual satisfaction, but not necessarily less masturbation.

**Table 6:** Predictors of the three masturbation and satisfaction clusters (LmS, HmD, LmD) in comparison with the reference group (HmS: High masturbation and Satisfied) in men (*n* = 900)

	Low/no masturbation Satisfied (LmS)		High masturbation Dissatisfied (HmD)		Low/no masturbation Dissatisfied (LmD)	
	AOR	95% CI	AOR	95% CI	AOR	95% CI
Sociodemographic characteristics						
Age	1.04^***^	1.03–1.06	1.01	0.99–1.03	1.03^***^	1.01–1.05
Education	0.87	0.67–1.12	1.34^*^	1.02–1.77	1.11	0.86–1.44
Religious	0.73	0.46–1.14	0.99	0.60–1.61	1.07	0.68–1.95
Relationship status^a^						
Unmarried^b^	1.07	0.59–1.92	0.62	0.34–1.16	0.21^***^	0.09–0.48
Separated/divorced^b^	0.33^*^	0.13–0.86	0.70	0.25–1.98	0.36	0.13–1.01
Widow/widower^b^	0.30	0.05–1.92	1.02	0.06–18.82	0.55	0.02–13.05
Psychological factor^c^						
Distress about sexual difficulties	1.18	0.92–1.51	1.57^***^	1.23–2.00	1.53^***^	1.19–1.95
Body image	0.84	0.67–1.06	1.35^**^	1.08–1.68	1.09	0.87–1.37
Genital self-image	1.00	0.81–1.23	1.24^*^	1.02–1.49	1.38^***^	1.13–1.69
Sexual behaviour^d^						
Number of sex partners (lifetime)	1.00	0.99–1.01	1.00	0.99–1.01	1.00	0.99–1.01
Frequency of pornography use	0.55^***^	0.48–0.62	1.43^***^	1.18–1.74	0.68^***^	0.59–0.78
Number of sexual experiences/desires	0.92	0.81–1.04	1.03	0.90–1.17	0.96	0.84–1.09
Intercourse frequency	1.30^***^	1.11–1.52	0.41^***^	0.33–0.50	0.49^***^	0.37–0.56

^*^p < 0.05. ^**^p < 0.01. ^***^p < 0.001

^a^ Reference category = married/cohabitant/registered partnership or in a current permanent relationship; ^b^ and currently not in a permanent relationship; ^c^ higher scores denote greater sexual distress/greater dissatisfaction; ^d^ higher scores denote higher frequency/numbers

#### Psychological Factors

Compared with the reference cluster (HmS), men who experienced distressing sexual problems were more likely to be dissatisfied with their sex life (AOR _HmD_ = 1.57; AOR _LmD_ = 1.53). Similarly, a more negative genital self-image was associated with being sexually dissatisfied (AOR _HmD_ = 1.24; AOR _LmD_ = 1.38), compared to participants in the reference cluster (HmS). Men with a negative body image were more likely to report high masturbation and being sexually dissatisfied (AOR _HmD_ = 1.35) than the reference group (HmS), implying that a negative body image is related to less sexual satisfaction, but not necessarily less masturbation.

#### Sexual Behavioral Factors

In terms of pornographic use, some interesting patterns of findings emerged. Men with frequent pornography use were less likely to fall into clusters characterized by low masturbation frequency (AOR _LmS_ = 0.55; AOR _LmD_ = 0.68) than the reference cluster (HmS). However, when comparing the two clusters characterized by high masturbation (HmS versus HmD), frequent pornography use was predictive of being sexually dissatisfied (AOR _HmD_ = 1.43). Regarding partnered sex, we found that men with higher intercourse frequency were more likely to report high masturbation and being sexually satisfied (HmS) than those belonging to a sexually dissatisfied cluster (AOR _HmD_ = 0.41; AOR _LmD_ = 0.49). Interestingly, when comparing the clusters characterized by high sexual satisfaction (HmS versus LmS), men with frequent intercourse were more likely to report no or low masturbation (AOR _LmS_ = 1.30).

## Discussion

Previous studies have focused on linear relationships between sexual satisfaction and masturbation frequency, without considering the possibility that women and men might vary in their masturbation–sexual satisfaction relationships. The clustering in this study revealed four groupings, with men’s and women’s sex life being characterized by either HmS, LmS, HmD, or LmD. Further, we assessed whether sociodemographic, psychological, and sexual behavioral factors predicted distinct masturbation–satisfaction patterns.

Two interesting patterns emerged. Psychological factors (sexual distress, body image and genital self-image) were more clearly related to sexual dissatisfaction, while age and sexual behavioral factors (pornography use, sexual experience and desires) were mainly linked to masturbation frequency. A possible reason for the fragmented findings may reflect that masturbatory behavior only partly contributes to a person’s overall sex life satisfaction. For example, Philippsohn and Hartmann (2009) found that masturbation was considerably less central in explaining women’s overall sexual satisfaction than sexual intercourse activity. Moreover, qualitative data from focus groups with 50 heterosexual men reveal that, compared to partnered sexual activities, masturbation was not fully integrated into men’s sense of being sexual (Janssen et al., 2008). These studies indicate that, although overlapping, sexual satisfaction from solitary and partnered sexuality might be different. Similarly, qualitative data from focus groups with 73 queer and heterosexual women showed that solitary and partnered sexual pleasure were largely distinct constructs, with only some overlap (Goldey et al., 2016). Future studies should therefore consider defining and measuring solitary and partnered sex life satisfaction as distinct concepts.

### A Compensatory or Complementary Pattern?

Women with higher sexual intercourse frequency were more likely to report high masturbation and satisfaction (HmS) than any other group (LmS, HmD, LmD). Also, more sexual experimentation among women was associated with more masturbation and satisfaction (HmS), compared to participants with LmS. Both findings support a complementary pattern for women, as it implies that frequent solo sex enhances partnered sex and is more widespread among adults with a sexualized personality pattern (e.g., increased sexual experimentation and desires) (Das et al., 2009).

Similar as in women, we found that men with higher intercourse frequency were more likely to be sexually satisfied (HmS), than those belonging to a sexually dissatisfied cluster (HmD or LmD). This is a finding that corresponds to previous studies that have found a positive relationship between partnered sex and sexual satisfaction (Brody & Costa, 2009; Byers & Rehman, 2014; Schoenfeld et al., 2017). However, when comparing men with high sexual satisfaction (HmS versus LmS), those with more partnered sex were more likely to report no or low masturbation (LmS). This finding supports a compensatory pattern in men, as it suggests that masturbation is regarded as unnecessary if one has highly satisfying and frequent sex with a partner (Regnerus et al., 2017). The gendered finding, revealing a compensatory pattern among men and a complementary pattern among women, is consistent with prior work supporting gender-specific models (Carvalheira & Leal, 2013; Fischer et al., 2022; Gerressu et al., 2008; Regnerus et al., 2017).

### Pornography Use Predict HmS

Another notable finding was that both women and men with frequent pornography use were more likely to report high masturbation and sexual satisfaction (HmS) than those belonging to a cluster characterized by no or low masturbation (LmS or LmD). This finding is similar to previous studies that have found a positive relationship between pornography use and masturbation (Baćak & Štulhofer, 2011; Carvalheira et al., 2015; Richters et al., 2014) and emphasizes that pornography functions as an aid for masturbation (Prause, 2019).

Apart from this, we found a link between pornography use, high masturbation, and sexual satisfaction in men (but not in women). When comparing men characterized by relatively high masturbation frequency (HmD vs. HmS), those with greater pornography use were more likely to report being sexually dissatisfied (HmD). This finding is consistent with a recent meta-analysis (Wright et al., 2017), which documented a negative association between men’s pornography use and sexual satisfaction, but no overall or global association between women’s pornography consumption and sexual satisfaction.

### Evaluative Factors Associated with Specific Masturbation-Satisfaction Typologies

Among both genders, a more negative body image was associated with being sexually dissatisfied (HmD in women and men; LmD in women), compared to participants in the reference cluster (HmS). This is consistent with previous evidence, implicating important links between body image and sexual satisfaction (Træen et al., 2016; Woertman & van den Brink, 2012). Interestingly, genital self-image was only linked to male cluster’s. In particular, a negative genital self-image was associated with being sexually dissatisfied (HmD and LmD), compared to the reference cluster (HmS). These findings echo those of a recent study, which revealed that when accounting for all body attitudes (body fat, genitals, muscularity, and height), only negative attitudes toward one’s own genitals were significantly associated with sexual dissatisfaction in men (van den Brink et al., 2018). The fact that men’s genitalia play an important role in defining masculinity in terms of appearance (e.g., penis size) and performance (e.g., erection) might explain the influences of men’s genital self-image on their sexual satisfaction.

Another central finding of the present study was that women and men who experienced distressing sexual problems were more likely to be dissatisfied with their sex life (HmD and LmD), compared to the reference cluster (HmS). This is in line with previous research indicating that sexual distress and sexual satisfaction are closely related (Stephenson & Meston, 2010).

### Links Between Sociodemographic Factors and Masturbation-Satisfaction Typologies

Some sociodemographic factors predicted specific masturbation-satisfaction typologies. Interestingly, although accounting for sexual intercourse frequency, relationship status remained an important predictor of high masturbation frequency and sexual satisfaction. Specifically, those who were married/cohabitant or in a registered partnership were less likely to report high masturbation and satisfaction than falling into a cluster characterized by LmS, HmD, LmD. This resembles findings of a recent large-scale study, which documented a negative association between being partnered and recent masturbation (Regnerus et al., 2017). As Regnerus et al. controlled for sexual frequency and sexual contentment, this was a surprising finding, providing “evidence that the effect of partnered status is not simply the effect of stable access to sex” (p. 2117).

For both women and men, higher education predicted a high masturbation frequency and sexual dissatisfaction (HmD). This finding dovetails with previous findings documenting a positive relationship between higher education and more masturbation (Gerressu et al., 2008; Kaminsky-Bayer, 2020; Kontula & Haavio-Mannila, 2003; Richters et al., 2014). However, previous research also seems to indicate that education does not play a major role in sexual satisfaction (Byers & Rehman, 2014). It is thus unclear why higher education was related to less sexual satisfaction among those who frequently masturbate. Finally, consistent with prior studies on age-related decreases in masturbation activity (Fischer et al., 2022; Lee et al., 2016; Schick et al., 2010a, 2010b), older age was related with a sex life characterized by low masturbation (LmS in women and men; LmD in men).

### Implications

The clusters characterized by no or low masturbation frequency and sexual satisfaction (HmS) were the largest clusters in both genders. This is interesting and may changes supposing the sexual scripts toward masturbation become more pronounced and positive in the future. The smallest clusters were those that included individuals dissatisfied with their sex life (HmD and LmD). To create a more masturbation-friendly society, future sexual health initiatives should focus on promoting masturbation and positive attitudes toward masturbation (Kontula & Haavio-Mannila, 2003).

### Study Limitations

Several limitations should be addressed. First, the item used to assess masturbation frequency lacked an explicit definition and contextualization of the term masturbation. As the measure does not solely refer to masturbation in unpartnered situations, it is possible that participants used varied definitions when responding to the question. Accordingly, we cannot rule out that some also referred to masturbation during sexual intercourse. However, recent evidence indicates that the common script for sexual self-pleasure incorporates solo rather than partnered masturbation (Kirschbaum & Pederson, 2018). Specifically, the absence of a partner and having an orgasm seem to be central aspects of labeling a sexual act as masturbation. Second, although the use of single-item indicators is standard practice and indicates good convergent validity with sexual satisfaction scales (Mark et al., 2014; Štulhofer et al., 2010), the psychometric properties of multiple-item scales are preferable. However, because many individuals seem to fill out online questionnaires on their mobile phones (in this study 51%), we had to prioritize single items to minimize response fatigue. Third, no information about attitudes toward masturbation and feelings associated with sexual self-pleasure was collected. Thus, third-variable problems cannot be ruled out. Assessing negative and positive perceptions of masturbation would have allowed for more differentiated clustering. Another limitation pertains the presumption of binary gender/sex in some questions. Also, because the results from this study are based on cross-sectional data, it is not feasible to draw any causal conclusions. Further, the possibility of social desirability bias and volunteer bias may affect our findings and limit the generalizability of the study findings (Boughner, 2010). A final limitation pertains to the low response rate. In the past decades, scientific research has experienced a steady decrease in participation rates (Galea & Tracy, 2007). This applies also to Norwegian surveys, where response rates have been declining from 63% in 1987, to 48% in 1992, 38% in 1997, 34% in 2002, and 23% in 2008 (Træen & Stigum, 2010). One reason for much higher refusal rates nowadays may be the growing number of instances people are asked to participate in studies (Galea & Tracy, 2007). Because this survey was carried out during the COVID-19-related lockdown, which was imposed on 12 March in Norway, it is possible that some Web Panel members were less receptive to participate in a study on sexual behavior. Moreover, it is uncertain how the COVID-19-related restrictions may have influenced our findings. Another explanation for the low response rate may pertain to the length of the questionnaire. According to Kantar, response rates for surveys drawn from the Gallup Panel vary between 46 and 51%. An estimated timeframe of 15–20 min for our survey was probably too long, especially because 51% of the respondents were answering on their mobile devices.

### Conclusion

To the best of our knowledge, this is the first study that provides a differentiated view of the relationship between masturbatory behavior and sexual satisfaction. Our findings suggest that the relation between masturbation frequency and sexual satisfaction does not necessarily develop in the same direction, and that there are different masturbation–sexual satisfaction typologies. Although this variability could be of substantial importance, it has generally been overlooked by the literature. Subsequent research is needed to further identify the nature of the relationship between masturbatory behavior and sexual satisfaction.
